# Different effects of air microembolism through patent foramen ovale in patients with migraine: A quantitative electroencephalogram case series

**DOI:** 10.3389/fneur.2022.1034714

**Published:** 2022-12-19

**Authors:** Raffaele Ornello, Matteo Spezialetti, Valeria Caponnetto, Ilaria Frattale, Monica Grappasonni, Francesca Pistoia, Giuseppe Placidi, Simona Sacco

**Affiliations:** ^1^Department of Biotechnological and Applied Clinical Sciences, University of L'Aquila, L'Aquila, Italy; ^2^Department of Information Engineering, Computer Science and Mathematics, University of L'Aquila, L'Aquila, Italy; ^3^Child Neurology and Neuropsychiatry Unit, Department of Systems Medicine, Tor Vergata University, Rome, Italy; ^4^Department of Neurology and Stroke Unit, SS Filippo e Nicola Hospital, Avezzano, L'Aquila, Italy; ^5^A2VI (Acquisition, Analysis, Visualization & Imaging Laboratory) Laboratory, Department of Life, Health and Environmental Sciences (MESVA), University of L'Aquila, L'Aquila, Italy

**Keywords:** migraine, patent foramen ovale, microembolism, migraine with aura, electroencephalogram

## Abstract

**Background:**

Literature suggests an association between patent foramen ovale (PFO) and migraine, mostly migraine with aura (MA). Previous data suggest that air microembolism through PFO can lead to bioelectrical abnormalities detectable at electroencephalogram (EEG) in patients with MA, thus suggesting a pathophysiological mechanism for the MA-PFO association. However, those data lack replication.

**Methods:**

Patients with MA or migraine without aura (MO) and large PFO underwent a 19-channel EEG recording before and after injection of air microbubbles. We compared EEG power before and after microbubble injection for each electrode location, for each frequency band (theta: 5–7 Hz; alpha: 8–12 Hz; beta: 13–30 Hz; lower gamma: 31–45 Hz), and for total global power (the average of EEG power at each location and frequency band).

**Results:**

We included 10 patients, four with MA and six with MO; six patients had medium-to-high migraine frequency (four or more monthly migraine days), while four had low frequency (one monthly migraine day). EEG power changes after air microembolism varied across patients. Considering the overall group, total global EEG power did not change; however, EEG power in the higher frequency ranges (beta and lower gamma) increased in patients with MA.

**Conclusions:**

We did not replicate the effects of air microembolism previously reported in patients with migraine. Aura status, migraine frequency, and medications might influence patients' response to microembolism. More refined EEG measurements are needed to clarify the dynamic role of PFO on migraine occurrence.

## 1. Introduction

A large body of literature showed an association between patent foramen ovale (PFO) and migraine, especially migraine with aura (MA), as pointed out in systematic reviews (1, 2). However, the functional significance of the association is still unclear. According to the most credited hypothesis, paradoxical embolism through PFO may induce cortical spreading depolarization (CSD) (3). Migraine aura is regarded as the clinical correlate of CSD (4). Therefore, at least theoretically, PFO closure should prevent MA by removing a source of cerebral embolism potentially inducing CSD events, However, pooled data from clinical trials suggest only a small benefit on to migraine from PFO closure which is mostly limited to those having MA (2, 5).

To evaluate the relevance of PFO in the genesis of migraine, it is interesting to assess the functional effects of paradoxical air embolism, which is commonly used at transcranial Doppler (TCD) to evaluate right-to-left cardiac shunt. A previous study showed that air microembolism through PFO produces a detectable effect on the activity of brain cortex, measured with quantitative electroencephalogram (qEEG), only in women with MA, while subjects with PFO and without migraine did not have any functional changes (6). As such, qEEG could theoretically provide a model to study the effect of microembolism in patients with migraine and thus the potential benefits of PFO closure. However, no subsequent replication studies are available.

The present case series aimed at investigating the effect of paradoxical air embolism on qEEG in patients with migraine and PFO.

## 2. Materials and methods

### 2.1. Patient selection

The present study was part of a broader case series of patients with MA or MO referring to the Headache Center of Avezzano-L'Aquila, Italy, diagnosed by expert physicians according to the International Classification of Headache Disorders (7), and studied with transcranial Doppler to detect PFO as a part of an experimental study. From February 2020 to October 2021, we selected 10 patients with MA or MO and a known large PFO (>20 microembolic signals or “curtain/shower effect” at transcranial Doppler) confirmed by previous examinations. To be included in the study, patients had to have active migraine, i.e., at least one episode per month throughout the previous 12 months. Both patients with and without migraine preventive treatments were included. Patients were excluded from the study if presenting major medical and/or psychiatric comorbidities.

### 2.2. Instrumental assessment

EEG recording was performed while patients were resting in a semirecumbent position in a quiet room, on a comfortable chair, with their eyes closed. A 19-channel EEG amplifier (EBNeuro, Italy) and the relative software were used for recordings and analyses. A standard electrode cap with 19 removable electrodes in accordance with the 10–20 system was used. Electrode impedances were kept at < 10 KΩ. Forehead reference and 4- to 45-Hz band-pass were applied. The sampling rate was 256 Hz.

Transcranial Doppler (TCD) was performed with an Acuson Sequoia 512 system (Siemens, Germany). All TCD evaluations were performed by the same operator (RO) to ensure standardization. To reduce interference with EEG recording, TCD recordings were performed at the internal carotid artery on the left submandibular window instead of the transtemporal window and with volume turned off.

An initial resting EEG recording was performed for 15 min. Afterwards, agitated saline (9 ml of saline and 1 ml of air) was injected *via* an antecubital intravenous catheter after the patient carried out a 5-s Valsalva maneuver to maximize microbubble transit. The time of microbubble transit through the left carotid artery was manually marked on EEG recordings. After microbubble transit, EEG recording was continued for 15 min. During the recording, patients' vigilance state was monitored by visual inspection of the EEG tracks throughout the registration.

### 2.3. qEEG analysis

EEG data were acquired for each of the 19 channels. Artifact removal was performed by decomposing the channels' signal into components through Independent Component Analysis (ICA) and automatically classifying brain and artifact components (8). The latter were discarded before projecting the signal back to the channels space. Signals of 3 min preceding and 3 min following microbubble injection, selected from the original 15-min periods, were considered for further analysis. Time-frequency analysis of preprocessed signals was then conducted by using Fast Fourier Transform (FFT) in order to obtain the raw power values for each frequency in the range 4–45 Hz, with a resolution of 1/180 Hz. For each band, selected with Hanning filters in the Fourier space, power was computed by averaging the power spectra over time and across frequencies of interest.

Pre-injection power spectra values were used as baseline. Following the indications from a previous study (6), the decibel change in power (Δ*P*) induced by microbubble injection was calculated for each electrode with the following formula: Δ*P* = 10^*^log (post-injection power/baseline power). Δ*P* was calculated for the total spectral range (4–45 Hz) and for each of the theta (4–7 Hz), alpha (8–13 Hz), beta (13–30 Hz), and lower gamma (31–45 Hz) bands. Frequencies <4 Hz and >45 Hz were not included in the analyses because of the possible occurrence of low-frequency noises and muscle activity, respectively. We also calculated a global total power by averaging the total power at all electrode locations.

### 2.4. Statistical analysis

Global total power, total power at each frequency range, and power at each electrode location were compared before and after microbubble injection for each subject by two-tailed *t*-test for independent variables. The same analyses were performed for subgroups of interest, according to the presence of aura and migraine frequency (low: one monthly day; medium-to-high: four or more monthly day), by averaging the power of each subject. Given the small sample size and the exploratory nature of our study, we conservatively set *p*-value threshold for significance at <0.001.

### 2.5. Ethical aspects

The study was approved by the Ethics Committee for the districts of L'Aquila and Teramo with protocol number 33/22. All patients signed an informed consent.

## 3. Results

Of the 10 subjects, one was a man and nine were women; the subjects' age ranged from 26 to 58 years; four patients had aura, while six patients did not. Seven patients were on preventive treatment, of whom three with monoclonal antibodies acting on the CGRP pathway, three with oral agents, and one with onabotulinumtoxinA (Table 1). Two patients reported headache soon after microbubble injection; none reported aura after microbubble injection.

**Table 1:** Characteristics of the included subjects and global power change in the different frequency bands.

**Subject**	**Gender**	**Age**	**Aura**	**Monthly migraine days**	**Ongoing prevention**	**Headache after procedure**	**Theta**		**Alpha**		**Beta**		**Lower gamma**		**Total**	
							**Δ*P***	***p*** **-Value**	**Δ*P***	***p*** **-Value**	**Δ*P***	***p*** **-Value**	**Δ*P***	***p*** **-Value**	**Δ*P***	***p*** **-Value**
1	Male	41	No	1	None	No	−0.35	0.281	+1.25	0.002	−0.23	0.224	+0.12	0.615	+0.38	0.085
2	Female	29	No	1	Erenumab 140 mg monthly	No	−0.38	0.420	+0.31	0.263	**+0.77**	**< 0.0001**	**+0.73**	**0.0006**	+0.29	0.104
3	Female	47	No	1	None	No	−14.90	0.018	−8.71	0.036	−8.49	0.071	−6.11	0.136	−10.14	0.031
4	Female	44	No	5	Erenumab 140 mg monthly	No	−0.90	0.050	**+1.69**	**< 0.0001**	**+3.43**	**< 0.0001**	**+4.30**	**< 0.0001**	**+1.21**	**< 0.0001**
5	Female	58	Yes	18	Galcanezumab 120 mg monthly	Yes	−0.97	0.003	+0.60	0.039	**+2.31**	**< 0.0001**	**+1.67**	**< 0.0001**	**+1.93**	**< 0.0001**
6	Female	34	Yes	14	OnabotulinumtoxinA 195 units quarterly	No	−0.78	0.762	−2.89	0.162	−3.36	0.377	−0.95	0.622	−2.21	0.394
7	Female	26	Yes	1	None	No	−0.65	0.280	+0.69	0.061	−0.54	0.024	−0.30	0.253	−0.03	0.929
8	Female	40	Yes	8	Topiramate 50 mg twice a day	Yes	+0.86	0.034	−0.20	0.576	**−1.17**	**< 0.0001**	**−2.93**	**< 0.0001**	−0.31	0.238
9	Female	49	No	4	Amitriptyline 20 mg once daily	No	+0.80	0.426	−0.62	0.543	−1.35	0.131	+0.84	0.209	+0.41	0.658
10	Female	52	No	6	Propranolol 40 mg daily	No	−0.13	0.675	**−1.30**	**0.0001**	+0.19	0.241	+0.67	0.007	**−0.82**	**0.0006**

Numbers in bold identify significant differences.

After microbubble injection, the EEG power changed differently for each subject and frequency band (Table 1). The global total EEG power did not change significantly in the overall group of patients (Table 2). However, in patients with MA or in those with medium-to-high migraine frequency, we found a significant power increase in the higher frequency spectrum (beta and lower gamma) that was not present in the other subgroups of interest (Table 2).

**Table 2:** Global power change in the overall group of patients and in subgroups of interest.

**Group**	***N***	**Theta**		**Alpha**		**Beta**		**Lower gamma**		**Total**	
		**Δ*P***	***p*** **-Value**	**Δ*P***	***p*** **-Value**	**Δ*P***	***p*** **-Value**	**Δ*P***	***p*** **-Value**	**Δ*P***	***p*** **-Value**
Overall	10	−0.63	0.208	−0.69	0.016	+1.36	0.001	**+1.43**	**< 0.001**	+0.35	0.269
With aura	4	−0.34	0.639	−0.51	0.268	**+1.52**	**< 0.001**	**+1.41**	**< 0.001**	+0.70	0.070
Without aura	6	−1.11	0.085	−1.07	0.006	−0.24	0.665	+0.58	0.032	−0.83	0.057
Medium-to-high frequency	6	−0.01	0.992	−0.86	0.029	**+1.75**	**< 0.001**	**+1.63**	**< 0.001**	+0.73	0.039
Low frequency	4	−1.99	0.015	−0.16	0.762	−1.54	0.053	−0.34	0.351	−1.06	0.090

Numbers in bold identify significant differences.

**Figure 1:**
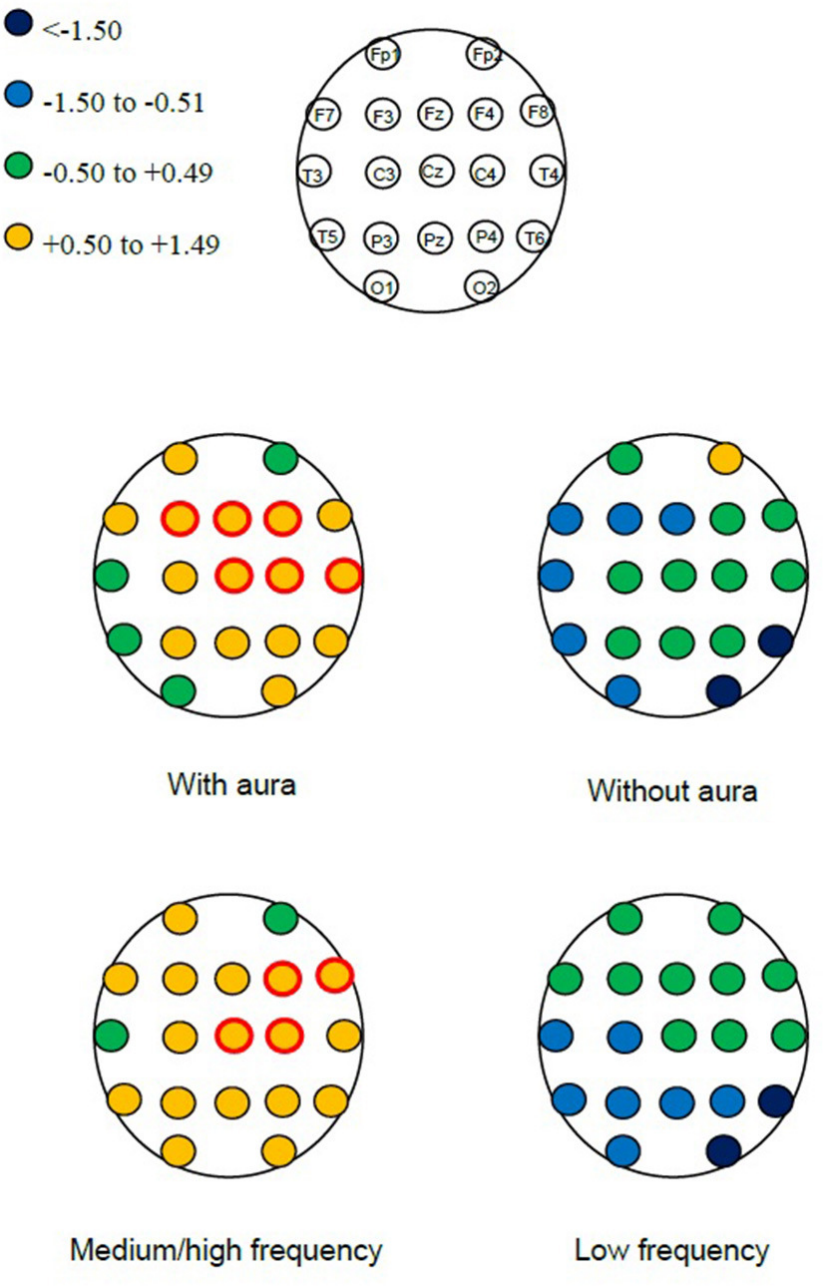
Detailed scheme of electroencephalographic power change (Δ*P*) after vs. before microbubble injection in the overall frequency spectrum (4–45 Hz) in subgroups of interest. Red circles identify changes with *p* < 0.001.

When considering each EEG electrode location, we found a significant power increase in central regions, driven by patients with MA and those with medium-to-high migraine frequency (Figure 1).

## 4. Discussion

In our case series of patients with migraine, we did not detect any significant change in total global EEG power after microbubble injection causing air microembolism through a PFO. However, we found a power increase in the higher frequency bands in patients with MA.

A previous study found that, among patients with PFO, those with MA showed an increased EEG power after microbubble injection that was not found in those without MA (6). A possible explanation of that finding is that patients with MA, compared with those without migraine, might have an increased cortical responsiveness to external stimuli, likely due to still unknown genetic factors influencing neurotransmitter levels and the neuronal metabolism. In our study including both patients with MA and MO, the increase in EEG power in high frequency bands was present only in the MA subgroup (Table 2, Figure 1), indicating the potentially high susceptibility of patients with MA to the effects of microembolism. However, we did not replicate the results of the previous study. We noticed little or no effect of air microembolism in the low-frequency bands (theta, alpha), while some effects were significant in the higher frequency range (beta, lower gamma); on the contrary, the previous study found a prominent power increase in the low frequency bands in patients with MA (6). Differences in the characteristics of included patients might explain this difference. Of note, in both studies only a minority of patients developed headache after microembolism, indicating that bioelectrical changes often lacked clinical correlates.

Cortical hyper-responsiveness is well-described in migraine (9–12) and might favor an increased response to any external stimulus brought to the cortex, including microembolism through PFO. Notably, cortical hyper-responsiveness related to migraine is typical of the visual cortex (9) while we found the highest EEG power changes in the central regions. Animal models showed that air microembolism can trigger cortical spreading depolarization events (3). However, the EEG patterns evoked by CSD in patients with MA still need to be reported in a standard fashion. We need a theoretical framework to obtain the expected EEG patterns induced by air microembolism and to know whether they can be attributed to cortical hyper-responsiveness and/or to cortical spreading depolarization.

Our findings suggest a different response to air microembolism driven by the presence of aura and by migraine frequency. However, we reported preliminary data from a restricted group of patients without previous sample size calculations. All subgroup analyses in our study are exploratory and should not be taken as conclusive. Besides, some physiological factors with a strong influence on migraine, such as those induced by the menstrual cycle, were not considered in this study. Some of the patients were taking migraine preventatives at the time of the study and we cannot exclude that this may have impacted on study findings. Our findings should be considered preliminary and need confirmation in larger populations. We selected 3-min periods which could have been too short to identify substantial alterations of the EEG signals. We also performed EEG spectral analysis without strict synchronization between the occurrence or number of microembolism and EEG measurements; this lack of synchronization might have added imprecision to our results. Further studies in the field should account for EEG dynamic changes over time. Future studies should also consider the transient increase in intracranial pressure associated to the Valsalva maneuver performed during transcranial Doppler. We performed our measurements with a 19-electrode EEG device; high-density EEG measurements or functional brain MRI would have added accurate anatomical details which were missing in this case series.

In conclusion, we did not replicate previous findings showing a relevant change in EEG spectral power after air microembolism; nevertheless, EEG power changes might vary according to the presence of aura and to migraine frequency. Our findings need to be confirmed in larger populations and more precise EEG measurements.

## Data availability statement

The raw data supporting the conclusions of this article will be made available by the authors, without undue reservation.

## Ethics statement

The studies involving human participants were reviewed and approved by Ethics Committee for the Districts of L'Aquila and Teramo. The patients/participants provided their written informed consent to participate in this study.

## Author contributions

RO and SS conceived and supervised the study. GP participated in setting up the protocol and revised the manuscript for intellectual content. MG participated in setting up the protocol and acquiring data. RO and MS acquired data and performed statistical analyses. IF and VC selected patients, performed the study procedures, and revised the manuscript for intellectual content. RO wrote the first draft of the manuscript. FP revised the manuscript for intellectual content. All authors contributed to the article and approved the submitted version.
